# Association between maternal fermented food consumption and infant sleep duration: The Japan Environment and Children's Study

**DOI:** 10.1371/journal.pone.0222792

**Published:** 2019-10-04

**Authors:** Narumi Sugimori, Kei Hamazaki, Kenta Matsumura, Haruka Kasamatsu, Akiko Tsuchida, Hidekuni Inadera, on behalf of the Japan Environment and Children’s Study Group

**Affiliations:** 1 Department of Public Health, Faculty of Medicine, University of Toyama, Toyama, Japan; 2 Toyama Regional Center for JECS, University of Toyama, Toyama, Japan; Universidade de Sao Paulo, BRAZIL

## Abstract

**Background:**

Evidence indicates that human circadian rhythm is affected by the intestinal microbiota, and establishment of the circadian rhythm begins during fetal development. However, the relationship between maternal fermented food intake and infant sleep duration has not been previously investigated. In this study, we examined whether dietary consumption of fermented food during pregnancy is associated with infant sleep duration at 1 year of age.

**Methods:**

This birth cohort study used data from a nationwide government-funded study called The Japan Environment and Children’s Study (JECS). After exclusions from a dataset comprising 104,065 JECS records, we evaluated 72,624 mother-child pairs where the child was 1 year old. We investigated the association between dietary intake of fermented foods during pregnancy and infant sleep duration of less than 11 h at 1 year of age.

**Results:**

Multivariable logistic regression showed that maternal intake of fermented food, especially *miso*, during the pregnancy was independently associated with reduced risk of infant sleep duration of less than 11 h.

**Conclusions:**

Further research, including interventional studies, is warranted to confirm the association between consumption of fermented foods during pregnancy and sufficient infant sleep duration.

**Trial registration:**

UMIN000030786.

## Introduction

Infants require sleep of sufficient duration and quality for healthy development. Sleep patterns commensurate with the child’s development change during the neonatal period and infancy. If these changes go beyond the normal range, developmental problems can occur, ranging from increased risk of obesity to neurodevelopmental disorders. For example, 6-year-olds who had short sleep duration in early infancy had a greater risk of hyperactivity than those who had adequate sleep duration [1]. Staying up late and short sleep duration have been linked to physical developmental issues, especially obesity, in 3-year-olds [2]. Also, sleep duration of less than 10.5 h before the age of 3 years was identified as a risk factor for infant obesity [3]. The importance of investigating the causes and effects of insufficient sleep duration in infancy are therefore clear and have been attracting researchers’ attention.

The dietary habits of expectant mothers are lifestyle factors that are generally recognized to affect fetuses and infants. Recently, food products containing probiotics have been actively promoted as part of the maternal diet and have garnered considerable interest [4]. Specifically, certain types of fermented food are thought to affect the intestinal microbiota and have been linked to the maintenance of maternal health [5, 6], or conversely the onset of illness, depending on the amount consumed [7, 8]. In addition, maternal microbiota and several other factors have been shown to affect the microbiota of infants [9–12]. Taken together with reports describing an association between infant microbiota and infant neurocognitive development [13, 14], the extent of maternal intake of fermented food may affect the normal development of fetuses, neonates, and infants, especially their sleep duration, which can be regarded as an indicator of normal development. However, the association between such intake and infant sleep duration has not been investigated on a sufficient scale in epidemiological studies.

In this study, we investigated whether maternal intake of fermented food during pregnancy affects infant sleep duration. We used logistic regression analysis of fixed data from mothers and their 1-year-old children obtained in a large-scale cohort study, the Japan Environment and Children’s Study (JECS).

## Materials and methods

### Study population

**Fig 1:**
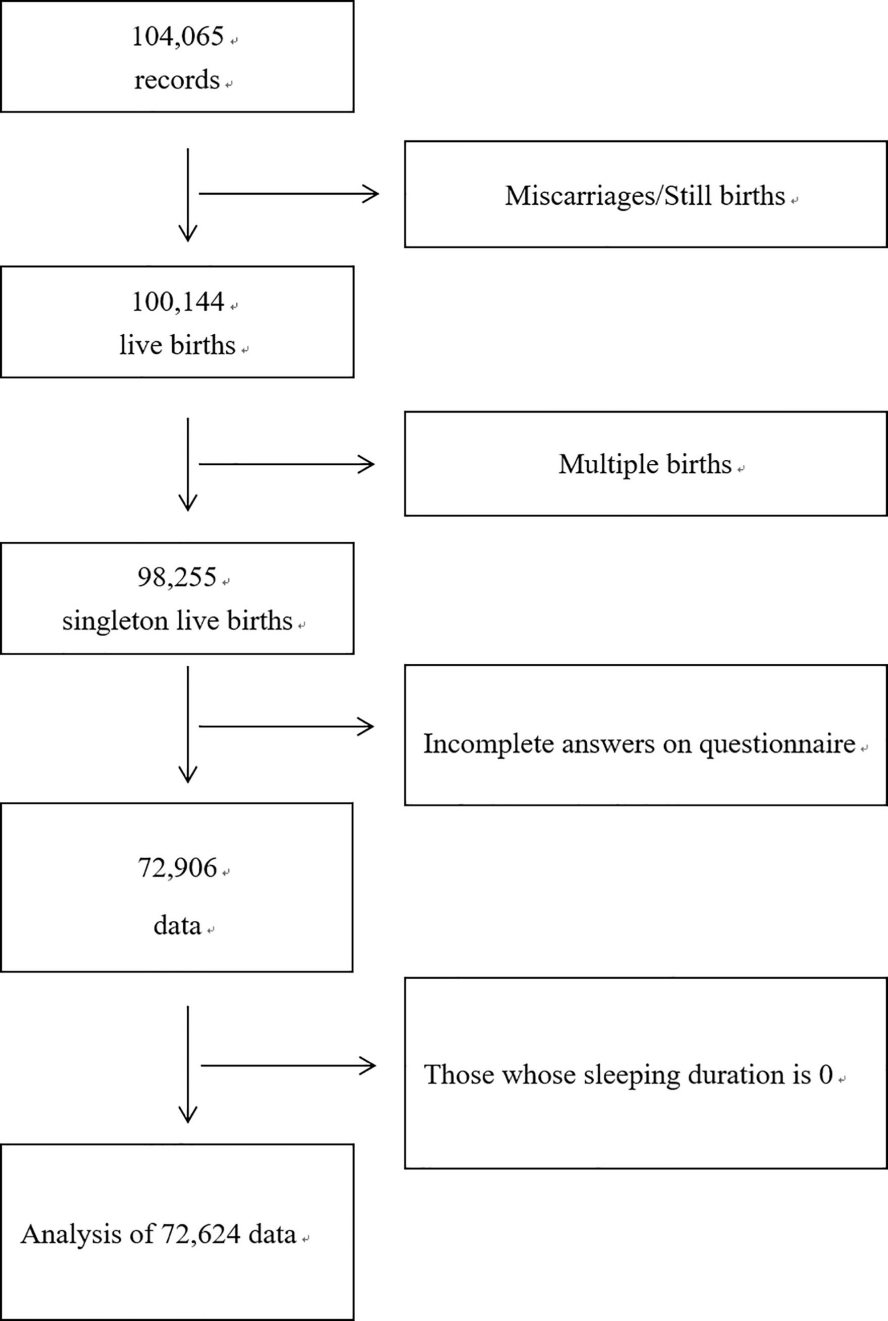
Flow diagram of the recruitment and exclusion process for participants.

The JECS protocol has been described in detail elsewhere [15, 16]. Briefly, the aim of the JECS, a nationwide government-funded birth cohort study, is to determine the impact of certain environmental factors on child health and development. JECS participants were women in the first trimester of pregnancy from 15 regions of Japan who were enrolled from January 2011 to March 2014 [15, 16]. The eligibility criteria for participants (expectant mothers) were as follows: 1) resident in a study area at the time of recruitment and expected to reside continually in Japan for the foreseeable future, 2) expected delivery date between 1 August 2011 and mid-2014, and 3) able to participate in the study without difficulty (i.e., able to understand Japanese and to complete the self-administered questionnaire). Excluded were expectant mothers residing outside a study area even if visiting cooperating healthcare providers within a study area [15]. The present study analyzed the jecs-an-20180131 dataset released in March 2018. The full dataset comprises 104,065 records obtained in a questionnaire survey of the participants. We excluded 3,921 and 1,889 records because of miscarriages/still births and multiple births, respectively (Fig 1), 24,424 records because of incomplete answers on the questionnaire, and 315 records for infants whose sleep duration was recorded as 0. The study protocol was approved by the Institutional Review Board on Epidemiological Studies of the Japanese Ministry of the Environment, and the ethics committees of all participating institutions: the National Institute for Environmental Studies, National Center for Child Health and Development, Hokkaido University, Sapporo Medical University, Asahikawa Medical College, Japanese Red Cross Hokkaido College of Nursing, Tohoku University, Fukushima Medical University, Chiba University, Yokohama City University, University of Yamanashi, Shinshu University, University of Toyama, Nagoya City University, Kyoto University, Doshisha University, Osaka University, Osaka Medical Center and Research Institute for Maternal and Child Health, Hyogo College of Medicine, Tottori University, Kochi University, University of Occupational and Environmental Health, Kyushu University, Kumamoto University, University of Miyazaki, and University of the Ryukyus. All participants provided written informed consent. The JECS is conducted in accordance with the Helsinki Declaration and all other national regulations.

### Data assessment

Dietary intake of fermented foods (*miso* soup, yogurt, cheese, and *natto* [Japanese fermented soybeans]) was determined using the Food Frequency Questionnaire, which is a self-administered, semi-quantitative instrument that has been validated for use in large-scale Japanese epidemiologic studies [17]. Participants were asked how often and how much they consumed each food type during the second and third trimesters (covering dietary intake after learning of the pregnancy). For *miso* soup, 6 frequency categories were used to record general consumption (almost none to every day), 9 frequency categories were used to record the frequency of daily consumption (< 1 time to ≥ 10 times), and 5 frequency categories were used for reporting the taste of *miso* (very bland to very strong). Daily intake of *miso* soup (g/day) was then calculated by multiplying the frequency of general consumption by the frequency of daily consumption and by the taste. For the other three fermented foods, yogurt, cheese, and *natto*, the standard portion size for each food type was categorized as small (50% smaller than standard), medium (same as standard), or large (50% larger than standard). Nine frequency categories for each item were used to record intake (< 1 time/month to ≥ 7 times/day). The daily intake of each of the three fermented foods was calculated by multiplying the frequency of consumption by the standard portion size and each was allocated into quartiles by their amount (g/day).

To measure infant sleep duration at 1 year after delivery, parents were asked on the questionnaire to indicate when their infant slept on the previous day. Parents marked the times when their infant was asleep by drawing lines through boxes indicating 30 min intervals from 12:00 am to 12:00 am the next day.

Covariates were adjusted for energy intake, maternal age, previous deliveries, body mass index at 1 month after delivery, maternal highest educational level, annual household income, marital status at 6 months after delivery, alcohol intake at 1 month after delivery, smoking status at 1 month after delivery, employment status at 1 year after delivery, infant sex, infant attendance at nursery, where the infant slept at night, birth weight, gestational period, presence of any disease, date (month) of birth, yogurt intake at 1 year old, and cheese intake at 1 year old.

### Statistical analysis

Unless otherwise stated, data are expressed as the mean ± standard deviation or median. Participants were categorized according to quartile for fermented food intake in order to estimate the risk of infant sleep duration shorter than 11 h. Sleep duration of 11–14 h of sleep in a 24-h period is recommended for 1-year-old infants by the United States National Sleep Foundation [18]. We therefore chose 11 h as the lower limit of appropriate sleep duration.

Odds ratios (ORs) and 95% confidence intervals (CIs) were calculated using logistic regression analysis, with the lowest quartile used as a reference. Adjusted ORs were calculated using the covariates mentioned in the previous section whereas crude ORs were calculated without use of these covariates. In tests for trend, categorical numbers were assigned to the quartile distributions for intake of each fermented food and were evaluated as continuous variables. Statistical significance was set at a 2-sided *p* value of < 0.05. Analyses were performed with SAS version 9.4 (SAS Institute Inc., Cary, NC).

## Results

Table 1 shows maternal characteristics according to quartile for *miso* intake during pregnancy. Those with higher *miso* intake were more likely to have high energy intake, to be multiparous, and to be a non-smoker.

**Table 1:** Characteristics according to quartile for *miso* intake during pregnancy in women (N = 72,624).

	Quartile for *miso* intake									
	Total		1 (low)		2		3		4(high)	
**Median intake of energy** ^**a**^	1,623		1,506		1,586		1,648		1,747	
**Age at delivery, years**	31.5		31.3		31.3		31.8		31.6	
**Previous deliveries, n (%)**										
Nullipara	29,896	(41.2)	8,677	(46.7)	6,493	(42.0)	7,980	(39.3)	6,746	(37.0)
Multipara	42,728	(58.8)	9,924	(53.4)	8,970	(58.0)	12,346	(60.7)	11,488	(63.0)
**BMI (kg/m**^**2**^**), n (%)**										
<18.5	3,593	(4.9)	940	(5.1)	761	(4.9)	1,045	(5.1)	847	(4.7)
18.5-<25	57,813	(79.6)	14,547	(78.2)	12,288	(79.5)	16,476	(81.1)	14,502	(79.5)
≥25	11,218	(15.4)	3,114	(16.7)	2,414	(15.6)	2,805	(13.8)	2,885	(15.8)
**Highest educational level, n (%)**										
Junior high school or high school	24,079	(33.2)	6,472	(34.8)	5,024	(32.5)	6,288	(30.9)	6,295	(34.5)
Technical junior college, technical/vocational college or associate degree	31,375	(43.2)	7,894	(42.4)	6,718	(43.5)	8,897	(43.8)	7,866	(43.1)
Bachelor’s degree or higher	17,170	(23.6)	4,235	(22.8)	3,721	(24.1)	5,141	(25.3)	4,073	(22.3)
**Annual household income (JPY), n (%)**										
<4 million	28,059	(38.6)	7,579	(40.8)	6,090	(39.4)	7,436	(36.6)	6,954	(38.1)
4–6 million	24,453	(33.7)	6,051	(32.5)	5,189	(33.6)	6,987	(34.4)	6,226	(34.2)
>6 million	20,112	(27.7)	4,971	(26.7)	4,184	(27.1)	5,903	(29.0)	5,054	(27.7)
**Marital status, n (%)**										
Married (including common law marriage)	71,598	(98.6)	18,238	(98.1)	15,239	(98.6)	20,106	(98.9)	18,015	(98.8)
Divorced or Widowed	490	(0.7)	167	(0.9)	102	(0.7)	108	(0.5)	113	(0.6)
Other	536	(0.7)	196	(1.1)	122	(0.8)	112	(0.6)	106	(0.6)
**Alcohol intake, n (%)**										
Never	66,560	(91.7)	16,937	(91.1)	14,146	(91.5)	18,696	(92.0)	16,781	(92.0)
Ex-drinker	3,201	(4.4)	835	(4.5)	696	(4.5)	890	(4.4)	780	(4.3)
1–3 times/month	1,971	(2.7)	554	(3.0)	444	(2.9)	526	(2.6)	447	(2.5)
≥ 1 time/week	892	(1.2)	275	(1.5)	177	(1.1)	214	(1.1)	226	(1.2)
**Smoking status, n (%)**										
Never	43,820	(60.3)	10,985	(59.1)	9,409	(60.9)	12,429	(61.2)	10,997	(60.3)
Did previously but quit before learning of pregnancy	16,861	(23.2)	4,245	(22.8)	3,569	(23.1)	4,786	(23.6)	4,261	(23.4)
Did previously but quit after learning of pregnancy	9,426	(13.0)	2,589	(13.9)	1,949	(12.6)	2,496	(12.3)	2,392	(13.1)
Currently smoking	2,517	(3.5)	782	(4.2)	536	(3.5)	615	(3.0)	584	(3.2)
**Employed, n (%)**										
No	37,404	(51.5)	9,496	(51.1)	8,175	(52.9)	10,637	(52.3)	9,096	(49.9)
Yes	35,220	(48.5)	9,105	(49.0)	7,288	(47.1)	9,689	(47.7)	9,138	(50.1)
**Infant sex, n (%)**										
Boy	37,109	(51.1)	9,485	(51.0)	7,963	(51.5)	10,422	(51.3)	9,239	(50.7)
Girl	35,515	(48.9)	9,116	(49.0)	7,500	(48.5)	9,904	(48.7)	8,995	(49.3)
**Nursery attendance, n (%)**										
No	52,804	(72.7)	13,399	(72.0)	11,373	(73.6)	14,850	(73.1)	13,182	(72.3)
Yes	19,820	(27.3)	5,202	(28.0)	4,090	(26.5)	5,476	(26.9)	5,052	(27.7)
**Location where infant sleeps at night, n (%)**										
In parent's bed	55,757	(76.8)	14,122	(75.9)	11,832	(76.5)	15,610	(76.8)	14,193	(77.8)
In baby bed in parents' bedroom	16,395	(22.6)	4,321	(23.2)	3,534	(22.9)	4,607	(22.7)	3,933	(21.6)
In baby bed in another room	389	(0.5)	137	(0.7)	83	(0.5)	87	(0.4)	82	(0.5)
Other	83	(0.1)	21	(0.1)	14	(0.1)	22	(0.1)	26	(0.1)
**Birth weight, g**	3,030		3,028		3,026		3,029		3,039	
**Gestational weeks**	39.3		39.3		39.3		39.3		39.2	
**Disease, n (%)**	13,775	(19.0)	3,668	(19.7)	3,051	(19.7)	3,698	(18.2)	3,358	(18.4)

^a^ Dietary intake between learning of pregnancy and second/third trimester.

BMI, body mass index

Unlike *miso*, three of the fermented foods—yogurt, cheese, and *natto*—showed similar characteristics according to quartile. Characteristics for yogurt are shown in Table 2; due to their similarity with yogurt, characteristics for cheese and *natto* are shown in S1 and S2 Tables, respectively. Pregnant women with higher intake of yogurt, cheese, and *natto* were more likely to be older, to have high energy intake, to have a higher education level, to have higher household income, to be employed, to be a non-smoker, and to be multiparous and they were less likely to send their infant to nursery.

**Table 2:** Characteristics according to quartile for yogurt intake during pregnancy in women (N = 72,624).

	Quartile for yogurt intake									
	Total		1 (low)		2		3		4(high)	
**Median intake of energy** ^**a**^	1,623		1,451		1,589		1,655		1,797	
**Age at delivery, years**	31.5		30.8		31.4		31.7		32.1	
**Previous deliveries, n (%)**										
Nullipara	29,896	(41.2)	7,221	(36.8)	5,883	(35.8)	6,771	(42.4)	10,021	(48.7)
Multipara	42,728	(58.8)	12,413	(63.2)	10,570	(64.2)	9,206	(57.6)	10,539	(51.3)
**BMI (kg/m**^**2**^**), n (%)**										
<18.5	3,593	(4.9)	899	(4.6)	765	(4.7)	797	(5.0)	1,132	(5.5)
18.5-<25	57,813	(79.6)	15,145	(77.1)	13,078	(79.5)	12,845	(80.4)	16,745	(81.4)
≥25	11,218	(15.4)	3,590	(18.3)	2,610	(15.9)	2,335	(14.6)	2,683	(13.1)
**Highest educational level, n (%)**										
Junior high school or high school	24,079	(33.2)	8,490	(43.2)	5,646	(34.3)	4,693	(29.4)	5,250	(25.5)
Technical junior college, technical/vocational college or associate degree	31,375	(43.2)	7,699	(39.2)	7,136	(43.4)	7,175	(44.9)	9,365	(45.6)
Bachelor’s degree or higher	17,170	(23.6)	3,445	(17.6)	3,671	(22.3)	4,109	(25.7)	5,945	(28.9)
**Annual household income (JPY), n (%)**										
<4 million	28,059	(38.6)	9,177	(46.7)	6,504	(39.5)	5,789	(36.2)	6,589	(32.1)
4–6 million	24,453	(33.7)	6,240	(31.8)	5,607	(34.1)	5,444	(34.1)	7,162	(34.8)
>6 million	20,112	(27.7)	4,217	(21.5)	4,342	(26.4)	4,744	(29.7)	6,809	(33.1)
**Marital status, n (%)**										
Married (including common law marriage)	71,598	(98.6)	19,254	(98.1)	16,226	(98.6)	15,779	(98.8)	20,339	(98.9)
Divorced or Widowed	490	(0.7)	192	(1.0)	99	(0.6)	94	(0.6)	105	(0.5)
Other	536	(0.7)	188	(1.0)	128	(0.8)	104	(0.7)	116	(0.6)
**Alcohol intake, n (%)**										
Never	66,560	(91.7)	17,657	(89.9)	15,035	(91.4)	14,719	(92.1)	19,149	(93.1)
Ex-drinker	3,201	(4.4)	982	(5.0)	740	(4.5)	683	(4.3)	796	(3.9)
1–3 times/month	1,971	(2.7)	634	(3.2)	479	(2.9)	416	(2.6)	442	(2.2)
≥ 1 time/week	892	(1.2)	361	(1.8)	199	(1.2)	159	(1.0)	173	(0.8)
**Smoking status, n (%)**										
Never	43,820	(60.3)	10,313	(52.5)	9,854	(59.9)	10,021	(62.7)	13,632	(66.3)
Did previously but quit before learning of pregnancy	16,861	(23.2)	4,689	(23.9)	3,835	(23.3)	3,701	(23.2)	4,636	(22.6)
Did previously but quit after learning of pregnancy	9,426	(13.0)	3,469	(17.7)	2,177	(13.2)	1,849	(11.6)	1,931	(9.4)
Currently smoking	2,517	(3.5)	1,163	(5.9)	587	(3.6)	406	(2.5)	361	(1.8)
**Employed, n (%)**										
No	37,404	(51.5)	10,117	(51.5)	8,429	(51.2)	8,128	(50.9)	10,730	(52.2)
Yes	35,220	(48.5)	9,517	(48.5)	8,024	(48.8)	7,849	(49.1)	9,830	(47.8)
**Infant sex, n (%)**										
Boy	37,109	(51.1)	9,965	(50.8)	8,386	(51.0)	8,253	(51.7)	10,505	(51.1)
Girl	35,515	(48.9)	9,669	(49.3)	8,067	(49.0)	7,724	(48.3)	10,055	(48.9)
**Nursery attendance, n (%)**										
No	52,804	(72.7)	13,798	(70.3)	11,895	(72.3)	11,669	(73.0)	15,442	(75.1)
Yes	19,820	(27.3)	5,836	(29.7)	4,558	(27.7)	4,308	(27.0)	5,118	(24.9)
**Location where infant sleeps at night, n (%)**										
In parent's bed	55,757	(76.8)	15,440	(78.6)	12,913	(78.5)	12,292	(76.9)	15,112	(73.5)
In baby bed in parents' bedroom	16,395	(22.6)	4,069	(20.7)	3,446	(20.9)	3,579	(22.4)	5,301	(25.8)
In baby bed in another room	389	(0.5)	100	(0.5)	78	(0.5)	85	(0.5)	126	(0.6)
Other	83	(0.1)	25	(0.1)	16	(0.1)	21	(0.1)	21	(0.1)
**Birth weight, g**	3,030		3,024		3,036		3,033		3,030	
**Gestational weeks**	39.3		39.3		39.3		39.3		39.3	
**Disease, n (%)**	13,775	(19.0)	3,712	(18.9)	3,112	(18.9)	3,062	(19.2)	3,889	(18.9)

^a^ Dietary intake between learning of pregnancy and second/third trimester.

BMI, body mass index

As for a possible selection bias, of the 104,065 records at baseline, 31,441 records were excluded for the 1-year analysis based on the exclusion criteria described above. Compared with excluded subjects, included subjects were more likely to eat yogurt and cheese, to be a nonsmoker, to have a higher education level, to have higher income, and to have babies with a heavier birth weight. There were no meaningful differences among the other covariates.

The ORs for infants not achieving the 11-h sleep duration target were evaluated based on the intake of probiotics by pregnant women in the second and third trimesters. The four probiotic food items evaluated were *miso*, yogurt, cheese, and *natto*. In the *miso* evaluation, ORs for inadequate sleep duration were significantly lower in infants with mothers in the upper (3rd and 4th) intake quartiles, and these associations were significant according to a trend test (Table 3).

**Table 3:** Odds ratios (95% confidence intervals) for 1-year-old infants for risk of sleeping less than 11 hours according to quartile for maternal intake of fermented food during pregnancy (N = 72,624).

	Quartiles for fermented food intake				*P*-value
	1 (low)	2	3	4(high)	for trend
**Median intake of *miso*, g/day** ^a^	10.0	32.1	88.4	225.0	
Total	18,601	15,463	20,326	18,234	
Cases	1,874	1,413	1,787	1,703	
Crude odds ratio	1.00	**0.90 (0.83–0.97)**	**0.86 (0.80–0.92)**	**0.92 (0.86–0.99)**	**0.005**
Adjusted odds ratio ^b^	1.00	**0.92 (0.85–0.98)**	**0.87 (0.82–0.94)**	**0.92 (0.86–0.99)**	**0.009**
**Median intake of yogurt, g/day** ^a^	8.0	25.7	60.0	120.0	
Total	19,634	16,453	15,977	20,560	
Cases	1,902	1,498	1,420	1,957	
Crude odds ratio	1.00	0.93 (0.87–1.00)	**0.91 (0.85–0.98)**	0.98 (0.92–1.05)	0.5
Adjusted odds ratio ^b^	1.00	0.96 (0.89–1.03)	0.93 (0.86–1.00)	0.99 (0.92–1.07)	0.8
**Median intake of cheese, g/day** ^a^	0.0	1.3	4.3	10.0	
Total	17,636	18,552	18,984	17,452	
Cases	1,748	1,709	1,679	1,641	
Crude odds ratio	1.00	0.92 (0.86–0.99)	0.88 (0.82–0.95)	0.94 (0.88–1.01)	**0.049**
Adjusted odds ratio ^b^	1.00	0.94 (0.88–1.01)	0.92 (0.85–0.99)	0.97 (0.90–1.05)	0.4
**Median intake of *natto*, g/day** ^a^	0.0	3.3	10.7	25.0	
Total	12,997	17,302	25,058	17,267	
Cases	1,273	1,630	2,257	1,617	
Crude odds ratio	1.00	0.96 (0.89–1.03)	**0.91 (0.85–0.98)**	0.95 (0.88–1.03)	0.11
Adjusted odds ratio ^b^	1.00	0.98 (0.90–1.06)	0.94 (0.88–1.02)	0.98 (0.90–1.06)	0.4

^a^ Dietary intake between learning of pregnancy and second/third trimester.

^b^ Covariates were adjusted for energy intake, maternal age, previous deliveries, BMI at 1 month after delivery, maternal highest educational level, annual household income, marital status at 6 months after delivery, alcohol intake at 1 month after delivery, smoking status at 1 month after delivery, employment status at 1 year after delivery, infant sex, infant attending nursery, location where infant slept at night, birth weight, gestational weeks, presence of any disease, date of birth, yogurt intake at 1 year old, and cheese intake at 1 year old.

## Discussion

In this study, we exploited an opportunity afforded by the JECS study to investigate sleep duration in 1-year-old infants, using questionnaire responses from 72,624 mothers for whom sufficient data were available. Our investigations showed that the risk of inadequate sleep duration (< 11 h) was significantly lower in the infants of mothers with high *miso* intake during the pregnancy.

The hypothesis for this study was that maternal intake of fermented food during pregnancy affects sleep duration in infants. We generated our hypothesis after reviewing a number of reports. Recent animal studies have shown that circadian rhythm is regulated by the liver and gut as well as the suprachiasmatic nucleus [19–21]. It has been reported that the intestinal microbiota has circadian rhythm [22, 23]. Intestinal microbiota is reported to be needed for the proper regulation of circadian rhythm, according to a comparison of the expression of the hepatic and mediobasal hypothalamic clock genes (*Bmal1* and *clock*) in intestinal-microbe–free mice and conventional mice; circadian rhythm was recognized in conventional mice but disrupted in the gut-microbe–free mice [24]. According to another report [25], patterns resembling a sleep-wake rhythm are observable through ultrasonographic monitoring of infant eyeball movement during the gestation period. This fetal sleep-wake cycle is surmised to be heavily influenced by maternal lifestyle factors such as diet and sleeping. Furthermore, maternal melatonin is known to have an effect on the fetus via placental transmission [26, 27]. Accordingly, maternal lifestyle is suggested to affect the healthy establishment of a circadian rhythm during fetal development.

Based on the above reports, we have developed the following, albeit hypothetical, line of reasoning. High fermented food intake during pregnancy has an effect on the intestinal microbiota of the expectant mother, and fetal circadian rhythm is also affected. Furthermore, intestinal microbiota migrates to the baby at the time of birth and produces changes in the baby’s intestinal microbiome [10]. We consider that this chain of causation leads to variation in sleep duration for the neonate or infant. In fact, although the pronounced rhythms in the sleep-wake cycle and in hormone secretion generally developed after 2 months of age [28], circadian rhythms have been observed for a number of different hormones and circulating factors as children age [29]. Accordingly, this association in older children must be studied. In this study, we did not directly investigate changes in the intestinal microbiota, and our study thus amounts to no more than supporting evidence at this time; nevertheless, our results provide indirect support for one part of the chain of causation referred to above.

This study had two main strengths: first, it was a large-scale study and, second, it used a validated questionnaire [17]. However, this study also has some limitations. We did not directly investigate changes in intestinal microbiota and this represents a weakness and limitation of the study, as stated above. Our reliance on maternal reporting of infant sleep duration is another limitation of this study. We observed that well-educated, employed, and higher-income women tended to have higher intake of fermented foods. To explain this, we surmise that these women are sufficiently knowledgeable about factors contributing to health and they therefore tend to select nutrient-rich options such as fermented foods more frequently than nutrient-deficient options like so-called junk food, and this health consciousness might affect the sleep duration of their infants. As such, our assumption that the results of our study reflected changes in intestinal microbiota remains a theory. Additional studies with different designs are needed to test this hypothesis, and they should examine indicators of changes in maternal intestinal flora and the condition of infant intestinal flora.

## Conclusion

In this study, we found that consumption of fermented food by expectant mothers was positively associated with infant sleep duration at 1 year of age, and we inferred that this association might be the result of changes in the gut microbiota of expectant mothers affected via their consumption of fermented food. To confirm our hypothesis, further research about the microbiota of women during pregnancy and that of their infants will be required.

## Supporting information

S1 Table Characteristics according to quartile for cheese intake during pregnancy in women (N = 72,624). ^a^ Dietary intake between learning of pregnancy and second/third trimester. BMI, body mass index.
(DOCX)

S2 Table Characteristics according to quartile for natto intake during pregnancy in women (N = 72,624). ^a^ Dietary intake between learning of pregnancy and second/third trimester. BMI, body mass index.
(DOCX)

## Abbreviations

CI: confidence interval
OR: odds ratio
JECS: Japan Environment and Children’s Study

## References

[1] Touchette E, Cote SM, Petit D, Liu X, Boivin M, Falissard B, et al Short nighttime sleep-duration and hyperactivity trajectories in early childhood. Pediatrics. 2009;124(5):e985–93. Epub 2009/10/21. 10.1542/peds.2008-2005 . 19841107

[2] Sekine M, Yamagami T, Handa K, Saito T, Nanri S, Kawaminami K, et al A dose-response relationship between short sleeping hours and childhood obesity: results of the Toyama Birth Cohort Study. Child Care Health Dev. 2002;28(2):163–70. Epub 2002/04/16. . 11952652 10.1046/j.1365-2214.2002.00260.x

[3] Reilly JJ, Armstrong J, Dorosty AR, Emmett PM, Ness A, Rogers I, et al Early life risk factors for obesity in childhood: cohort study. BMJ. 2005;330(7504):1357 Epub 2005/05/24. 10.1136/bmj.38470.670903.E0 15908441 PMC558282

[4] Sullivan A, Nord CE. Probiotics and gastrointestinal diseases. J Intern Med. 2005;257(1):78–92. Epub 2004/12/21. 10.1111/j.1365-2796.2004.01410.x . 15606379

[5] Butel MJ. Probiotics, gut microbiota and health. Med Mal Infect. 2014;44(1):1–8. Epub 2013/12/03. 10.1016/j.medmal.2013.10.002 . 24290962

[6] D R. The effect of probiotic supplementation on self-reported sleep quality. 2016.

[7] Gille D, Schmid A, Walther B, Vergeres G. Fermented Food and Non-Communicable Chronic Diseases: A Review. Nutrients. 2018;10(4). Epub 2018/04/05. 10.3390/nu10040448 29617330 PMC5946233

[8] Mantaring J, Benyacoub J, Destura R, Pecquet S, Vidal K, Volger S, et al Effect of maternal supplement beverage with and without probiotics during pregnancy and lactation on maternal and infant health: a randomized controlled trial in the Philippines. BMC Pregnancy Childbirth. 2018;18(1):193 Epub 2018/06/02. 10.1186/s12884-018-1828-8 29855271 PMC5984298

[9] Sanz Y. Gut microbiota and probiotics in maternal and infant health. Am J Clin Nutr. 2011;94(6 Suppl):2000S–5S. Epub 2011/05/06. 10.3945/ajcn.110.001172 . 21543533

[10] Backhed F, Roswall J, Peng Y, Feng Q, Jia H, Kovatcheva-Datchary P, et al Dynamics and Stabilization of the Human Gut Microbiome during the First Year of Life. Cell Host Microbe. 2015;17(5):690–703. Epub 2015/05/15. 10.1016/j.chom.2015.04.004 . 25974306

[11] Nuriel-Ohayon M, Neuman H, Koren O. Microbial Changes during Pregnancy, Birth, and Infancy. Front Microbiol. 2016;7:1031 Epub 2016/07/30. 10.3389/fmicb.2016.01031 27471494 PMC4943946

[12] Stewart CJ, Ajami NJ, O'Brien JL, Hutchinson DS, Smith DP, Wong MC, et al Temporal development of the gut microbiome in early childhood from the TEDDY study. Nature. 2018;562(7728):583–8. Epub 2018/10/26. 10.1038/s41586-018-0617-x 30356187 PMC6415775

[13] Yang I, Corwin EJ, Brennan PA, Jordan S, Murphy JR, Dunlop A. The Infant Microbiome: Implications for Infant Health and Neurocognitive Development. Nurs Res. 2016;65(1):76–88. Epub 2015/12/15. 10.1097/NNR.0000000000000133 26657483 PMC4681407

[14] Carlson AL, Xia K, Azcarate-Peril MA, Goldman BD, Ahn M, Styner MA, et al Infant Gut Microbiome Associated With Cognitive Development. Biol Psychiatry. 2018;83(2):148–59. Epub 2017/08/11. 10.1016/j.biopsych.2017.06.021 28793975 PMC5724966

[15] Kawamoto T, Nitta H, Murata K, Toda E, Tsukamoto N, Hasegawa M, et al Rationale and study design of the Japan environment and children's study (JECS). BMC public health. 2014;14:25 Epub 2014/01/15. 10.1186/1471-2458-14-25 24410977 PMC3893509

[16] Michikawa T, Nitta H, Nakayama SF, Yamazaki S, Isobe T, Tamura K, et al Baseline Profile of Participants in the Japan Environment and Children's Study (JECS). J Epidemiol. 2018;28(2):99–104. Epub 2017/11/03. 10.2188/jea.JE20170018 29093304 PMC5792233

[17] Yokoyama Y, Takachi R, Ishihara J, Ishii Y, Sasazuki S, Sawada N, et al Validity of Short and Long Self-Administered Food Frequency Questionnaires in Ranking Dietary Intake in Middle-Aged and Elderly Japanese in the Japan Public Health Center-Based Prospective Study for the Next Generation (JPHC-NEXT) Protocol Area. J Epidemiol. 2016;26(8):420–32. Epub 2016/04/12. 10.2188/jea.JE20150064 27064130 PMC4967663

[18] Hirshkowitz M, Whiton K, Albert SM, Alessi C, Bruni O, DonCarlos L, et al National Sleep Foundation's updated sleep duration recommendations: final report. Sleep Health. 2015;1(4):233–43. Epub 2015/12/01. 10.1016/j.sleh.2015.10.004 . 29073398

[19] Konturek PC, Brzozowski T, Konturek SJ. Gut clock: implication of circadian rhythms in the gastrointestinal tract. J Physiol Pharmacol. 2011;62(2):139–50. Epub 2011/06/16. . 21673361

[20] Pan X. Clock is important for food and circadian regulation of macronutrient absorption in mice. Journal of Lipid Research. 2009;50:1800–13. 10.1194/jlr.M900085-JLR200 19387090 PMC2724783

[21] Scheving LA. Biological clocks and the digestive system. Gastroenterology. 2000;119(2):536–49. Epub 2000/08/10. 10.1053/gast.2000.9305 . 10930389

[22] Thaiss CA, Zeevi D, Levy M, Zilberman-Schapira G, Suez J, Tengeler AC, et al Transkingdom control of microbiota diurnal oscillations promotes metabolic homeostasis. Cell. 2014;159(3):514–29. Epub 2014/11/25. 10.1016/j.cell.2014.09.048 . 25417104

[23] Liang X. Rhythmicity of the intestinal microbiota is regulated by gender and the host circadian clock. PNAS. 2015:10479–84. 10.1073/pnas.1501305112 26240359 PMC4547234

[24] Leone V, Gibbons SM, Martinez K, Hutchison AL, Huang EY, Cham CM, et al Effects of diurnal variation of gut microbes and high-fat feeding on host circadian clock function and metabolism. Cell Host Microbe. 2015;17(5):681–9. Epub 2015/04/22. 10.1016/j.chom.2015.03.006 25891358 PMC4433408

[25] Okawa H, Morokuma S, Maehara K, Arata A, Ohmura Y, Horinouchi T, et al Eye movement activity in normal human fetuses between 24 and 39 weeks of gestation. PLoS One. 2017;12(7):e0178722 Epub 2017/07/13. 10.1371/journal.pone.0178722 28700709 PMC5507482

[26] Bisanti L, Olsen J, Basso O, Thonneau P, Karmaus W. Shift work and subfecundity: a European multicenter study. European Study Group on Infertility and Subfecundity. J Occup Environ Med. 1996;38(4):352–8. Epub 1996/04/01. 10.1097/00043764-199604000-00012 . 8925318

[27] Mendez N, Abarzua-Catalan L, Vilches N, Galdames HA, Spichiger C, Richter HG, et al Timed maternal melatonin treatment reverses circadian disruption of the fetal adrenal clock imposed by exposure to constant light. PLoS One. 2012;7(8):e42713 Epub 2012/08/23. 10.1371/journal.pone.0042713 22912724 PMC3418288

[28] Rivkees SA. Developing circadian rhythmicity in infants. Pediatrics. 2003;112(2):373–81. Epub 2003/08/05. 10.1542/peds.112.2.373 . 12897290

[29] Hellbruegge T, Lange JE, Stehr K, Rutenfranz J. Circadian Periodicity of Physiological Functions in Different Stages of Infancy and Childhood. Annals of the New York Academy of Sciences. 1964;117:361–73. Epub 1964/09/10. 10.1111/j.1749-6632.1964.tb48193.x . 14196654

