# Supplementary material for: Association between maternal fermented food consumption and infant sleep duration: The Japan Environment and Children's Study
Source: PLoS One. 2019 Oct 4;14(10):e0222792. doi: 10.1371/journal.pone.0222792 (PMC6777830; doi:10.1371/journal.pone.0222792)
Supplement: S1 Table — a Dietary intake between learning of pregnancy and second/third trimester. BMI, body mass index. (DOCX) [file pone.0222792.s001.docx]

**S1 Table. Characteristics according to quartile for cheese intake during pregnancy in women (N=72,624)**

|  | Quartiles for cheese intake | | | | | | | | | |
| --- | --- | --- | --- | --- | --- | --- | --- | --- | --- | --- |
|  | Total | | 1 (low) | | 2 | | 3 | | 4(high) | |
| **Median intake of energy ^a^** | 1,623 | | 1,427 | | 1,530 | | 1,672 | | 1,882 | |
| **Age at delivery, years** | 31.5 | | 30.8 | | 31.2 | | 31.8 | | 32.2 | |
| **Previous deliveries, n (%)** |  |  |  |  |  |  |  |  |  |  |
| Nullipara | 29,896 | ( 41.2 ) | 7,936 | ( 45.0 ) | 7,981 | ( 43.0 ) | 7,407 | ( 39.0 ) | 6,572 | ( 37.7 ) |
| Multipara | 42,728 | ( 58.8 ) | 9,700 | ( 55.0 ) | 10,571 | ( 57.0 ) | 11,577 | ( 61.0 ) | 10,880 | ( 62.3 ) |
| **BMI (kg/m^2^), n (%)** |  |  |  |  |  |  |  |  |  |  |
| <18.5 | 3,593 | ( 4.9 ) | 895 | ( 5.1 ) | 857 | ( 4.6 ) | 947 | ( 5.0 ) | 894 | ( 5.1 ) |
| 18.5-<25 | 57,813 | ( 79.6 ) | 13,628 | ( 77.3 ) | 14,714 | ( 79.3 ) | 15,378 | ( 81.0 ) | 14,093 | ( 80.8 ) |
| ≥25 | 11,218 | ( 15.4 ) | 3,113 | ( 17.7 ) | 2,981 | ( 16.1 ) | 2,659 | ( 14.0 ) | 2,465 | ( 14.1 ) |
| **Highest educational level, n (%)** |  |  |  |  |  |  |  |  |  |  |
| Junior high school or high school | 24,079 | ( 33.2 ) | 7,305 | ( 41.4 ) | 6,501 | ( 35.0 ) | 5,600 | ( 29.5 ) | 4,673 | ( 26.8 ) |
| Technical junior college, technical/vocational college or associate degree | 31,375 | ( 43.2 ) | 7,247 | ( 41.1 ) | 7,979 | ( 43.0 ) | 8,418 | ( 44.3 ) | 7,731 | ( 44.3 ) |
| Bachelor’s degree or higher | 17,170 | ( 23.6 ) | 3,084 | ( 17.5 ) | 4,072 | ( 22.0 ) | 4,966 | ( 26.2 ) | 5,048 | ( 28.9 ) |
| **Annual household income (JPY), n (%)** |  |  |  |  |  |  |  |  |  |  |
| <4 million | 28,059 | ( 38.6 ) | 7,970 | ( 45.2 ) | 7,268 | ( 39.2 ) | 6,887 | ( 36.3 ) | 5,934 | ( 34.0 ) |
| 4-6 million | 24,453 | ( 33.7 ) | 5,475 | ( 31.0 ) | 6,272 | ( 33.8 ) | 6,566 | ( 34.6 ) | 6,140 | ( 35.2 ) |
| >6 million | 20,112 | ( 27.7 ) | 4,191 | ( 23.8 ) | 5,012 | ( 27.0 ) | 5,531 | ( 29.1 ) | 5,378 | ( 30.8 ) |
| **Marital status, n (%)** |  |  |  |  |  |  |  |  |  |  |
| Married (including common law marriage) | 71,598 | ( 98.6 ) | 17,270 | ( 97.9 ) | 18,286 | ( 98.6 ) | 18,773 | ( 98.9 ) | 17,269 | ( 99.0 ) |
| Divorced or Widowed | 490 | ( 0.7 ) | 160 | ( 0.9 ) | 142 | ( 0.8 ) | 102 | ( 0.5 ) | 86 | ( 0.5 ) |
| Other | 536 | ( 0.7 ) | 206 | ( 1.2 ) | 124 | ( 0.7 ) | 109 | ( 0.6 ) | 97 | ( 0.6 ) |
| **Alcohol intake, n (%)** |  |  |  |  |  |  |  |  |  |  |
| Never | 66,560 | ( 91.7 ) | 16,240 | ( 92.1 ) | 16,994 | ( 91.6 ) | 17,403 | ( 91.7 ) | 15,923 | ( 91.2 ) |
| Ex-drinker | 3,201 | ( 4.4 ) | 711 | ( 4.0 ) | 804 | ( 4.3 ) | 886 | ( 4.7 ) | 800 | ( 4.6 ) |
| 1-3 times/month | 1,971 | ( 2.7 ) | 453 | ( 2.6 ) | 514 | ( 2.8 ) | 491 | ( 2.6 ) | 513 | ( 2.9 ) |
| ≥ 1 time/week | 892 | ( 1.2 ) | 232 | ( 1.3 ) | 240 | ( 1.3 ) | 204 | ( 1.1 ) | 216 | ( 1.2 ) |
| **Smoking status, n (%)** |  |  |  |  |  |  |  |  |  |  |
| Never | 43,820 | ( 60.3 ) | 9,836 | ( 55.8 ) | 11,030 | ( 59.5 ) | 11,964 | ( 63.0 ) | 10,990 | ( 63.0 ) |
| Did previously but quit before learning of pregnancy | 16,861 | ( 23.2 ) | 3,962 | ( 22.5 ) | 4,307 | ( 23.2 ) | 4,359 | ( 23.0 ) | 4,233 | ( 24.3 ) |
| Did previously but quit after learning of pregnancy | 9,426 | ( 13.0 ) | 2,935 | ( 16.6 ) | 2,540 | ( 13.7 ) | 2,171 | ( 11.4 ) | 1,780 | ( 10.2 ) |
| Currently smoking | 2,517 | ( 3.5 ) | 903 | ( 5.1 ) | 675 | ( 3.6 ) | 490 | ( 2.6 ) | 449 | ( 2.6 ) |
| **Employed, n (%)** |  |  |  |  |  |  |  |  |  |  |
| No | 37,404 | ( 51.5 ) | 8,784 | ( 49.8 ) | 9,340 | ( 50.3 ) | 9,913 | ( 52.2 ) | 9,367 | ( 53.7 ) |
| Yes | 35,220 | ( 48.5 ) | 8,852 | ( 50.2 ) | 9,212 | ( 49.7 ) | 9,071 | ( 47.8 ) | 8,085 | ( 46.3 ) |
| **Infant sex, n (%)** |  |  |  |  |  |  |  |  |  |  |
| Boy | 37,109 | ( 51.1 ) | 9,025 | ( 51.2 ) | 9,390 | ( 50.6 ) | 9,857 | ( 51.9 ) | 8,837 | ( 50.6 ) |
| Girl | 35,515 | ( 48.9 ) | 8,611 | ( 48.8 ) | 9,162 | ( 49.4 ) | 9,127 | ( 48.1 ) | 8,615 | ( 49.4 ) |
| **Nursery attendance, n (%)** |  |  |  |  |  |  |  |  |  |  |
| No | 52,804 | ( 72.7 ) | 12,341 | ( 70.0 ) | 13,317 | ( 71.8 ) | 14,059 | ( 74.1 ) | 13,087 | ( 75.0 ) |
| Yes | 19,820 | ( 27.3 ) | 5,295 | ( 30.0 ) | 5,235 | ( 28.2 ) | 4,925 | ( 25.9 ) | 4,365 | ( 25.0 ) |
| **Location where infant sleeps at night, n (%)** |  |  |  |  |  |  |  |  |  |  |
| In parent's bed | 55,757 | ( 76.8 ) | 13,442 | ( 76.2 ) | 14,149 | ( 76.3 ) | 14,663 | ( 77.2 ) | 13,503 | ( 77.4 ) |
| In baby bed in parents' bedroom | 16,395 | ( 22.6 ) | 4,077 | ( 23.1 ) | 4,296 | ( 23.2 ) | 4,196 | ( 22.1 ) | 3,826 | ( 21.9 ) |
| In baby bed in another room | 389 | ( 0.5 ) | 89 | ( 0.5 ) | 91 | ( 0.5 ) | 101 | ( 0.5 ) | 108 | ( 0.6 ) |
| Other | 83 | ( 0.1 ) | 28 | ( 0.2 ) | 16 | ( 0.1 ) | 24 | ( 0.1 ) | 15 | ( 0.1 ) |
| **Birth weight, g** | 3,030 | | 3,019 | | 3,031 | | 3,036 | | 3,035 | |
| **Gestational weeks** | 39.3 | | 39.3 | | 39.3 | | 39.3 | | 39.3 | |
| **Disease, n (%)** | 13,775 | ( 19.0 ) | 3,315 | ( 18.8 ) | 3,474 | ( 18.7 ) | 3,654 | ( 19.3 ) | 3,332 | ( 19.1 ) |

^a^ Dietary intake between learning of pregnancy and second/third trimester.

BMI, body mass index
