# Supplementary material for: Association between maternal fermented food consumption and infant sleep duration: The Japan Environment and Children's Study
Source: PLoS One. 2019 Oct 4;14(10):e0222792. doi: 10.1371/journal.pone.0222792 (PMC6777830; doi:10.1371/journal.pone.0222792)
Supplement: S2 Table — a Dietary intake between learning of pregnancy and second/third trimester. BMI, body mass index. (DOCX) [file pone.0222792.s002.docx]

**S2 Table. Characteristics according to quartile for *natto* intake during pregnancy in women (N=72,624)**

|  | Quartiles for natto intake | | | | | | | | | |
| --- | --- | --- | --- | --- | --- | --- | --- | --- | --- | --- |
|  | Total | | 1 (low) | | 2 | | 3 | | 4(high) | |
| **Median intake of energy ^a^** | 1,623 | | 1,467 | | 1,487 | | 1,643 | | 1,856 | |
| **Age at delivery, years** | 31.5 | | 31.2 | | 31.1 | | 31.6 | | 32.1 | |
| **Previous deliveries, n (%)** |  |  |  |  |  |  |  |  |  |  |
| Nullipara | 29,896 | ( 41.2 ) | 5,813 | ( 44.7 ) | 7,423 | ( 42.9 ) | 9,808 | ( 39.1 ) | 6,852 | ( 39.7 ) |
| Multipara | 42,728 | ( 58.8 ) | 7,184 | ( 55.3 ) | 9,879 | ( 57.1 ) | 15,250 | ( 60.9 ) | 10,415 | ( 60.3 ) |
| **BMI (kg/m^2^), n (%)** |  |  |  |  |  |  |  |  |  |  |
| <18.5 | 3,593 | ( 4.9 ) | 660 | ( 5.1 ) | 787 | ( 4.6 ) | 1,235 | ( 4.9 ) | 911 | ( 5.3 ) |
| 18.5-<25 | 57,813 | ( 79.6 ) | 10,162 | ( 78.2 ) | 13,765 | ( 79.6 ) | 20,105 | ( 80.2 ) | 13,781 | ( 79.8 ) |
| ≥25 | 11,218 | ( 15.4 ) | 2,175 | ( 16.7 ) | 2,750 | ( 15.9 ) | 3,718 | ( 14.8 ) | 2,575 | ( 14.9 ) |
| **Highest educational level, n (%)** |  |  |  |  |  |  |  |  |  |  |
| Junior high school or high school | 24,079 | ( 33.2 ) | 4,613 | ( 35.5 ) | 6,065 | ( 35.1 ) | 8,131 | ( 32.5 ) | 5,270 | ( 30.5 ) |
| Technical junior college, technical/vocational college or associate degree | 31,375 | ( 43.2 ) | 5,503 | ( 42.3 ) | 7,366 | ( 42.6 ) | 10,858 | ( 43.3 ) | 7,648 | ( 44.3 ) |
| Bachelor’s degree or higher | 17,170 | ( 23.6 ) | 2,881 | ( 22.2 ) | 3,871 | ( 22.4 ) | 6,069 | ( 24.2 ) | 4,349 | ( 25.2 ) |
| **Annual household income (JPY), n (%)** |  |  |  |  |  |  |  |  |  |  |
| <4 million | 28,059 | ( 38.6 ) | 5,421 | ( 41.7 ) | 6,914 | ( 40.0 ) | 9,371 | ( 37.4 ) | 6,353 | ( 36.8 ) |
| 4-6 million | 24,453 | ( 33.7 ) | 4,225 | ( 32.5 ) | 5,759 | ( 33.3 ) | 8,584 | ( 34.3 ) | 5,885 | ( 34.1 ) |
| >6 million | 20,112 | ( 27.7 ) | 3,351 | ( 25.8 ) | 4,629 | ( 26.8 ) | 7,103 | ( 28.4 ) | 5,029 | ( 29.1 ) |
| **Marital status, n (%)** |  |  |  |  |  |  |  |  |  |  |
| Married (including common law marriage) | 71,598 | ( 98.6 ) | 12,745 | ( 98.1 ) | 17,049 | ( 98.5 ) | 24,733 | ( 98.7 ) | 17,071 | ( 98.9 ) |
| Divorced or Widowed | 490 | ( 0.7 ) | 126 | ( 1.0 ) | 124 | ( 0.7 ) | 158 | ( 0.6 ) | 82 | ( 0.5 ) |
| Other | 536 | ( 0.7 ) | 126 | ( 1.0 ) | 129 | ( 0.8 ) | 167 | ( 0.7 ) | 114 | ( 0.7 ) |
| **Alcohol intake, n (%)** |  |  |  |  |  |  |  |  |  |  |
| Never | 66,560 | ( 91.7 ) | 11,952 | ( 92.0 ) | 15,810 | ( 91.4 ) | 22,920 | ( 91.5 ) | 15,878 | ( 92.0 ) |
| Ex-drinker | 3,201 | ( 4.4 ) | 587 | ( 4.5 ) | 773 | ( 4.5 ) | 1,137 | ( 4.5 ) | 704 | ( 4.1 ) |
| 1-3 times/month | 1,971 | ( 2.7 ) | 305 | ( 2.4 ) | 503 | ( 2.9 ) | 697 | ( 2.8 ) | 466 | ( 2.7 ) |
| ≥ 1 time/week | 892 | ( 1.2 ) | 153 | ( 1.2 ) | 216 | ( 1.3 ) | 304 | ( 1.2 ) | 219 | ( 1.3 ) |
| **Smoking status, n (%)** |  |  |  |  |  |  |  |  |  |  |
| Never | 43,820 | ( 60.3 ) | 7,920 | ( 60.9 ) | 10,192 | ( 58.9 ) | 15,067 | ( 60.1 ) | 10,641 | ( 61.6 ) |
| Did previously but quit before learning of pregnancy | 16,861 | ( 23.2 ) | 2,633 | ( 20.3 ) | 3,971 | ( 23.0 ) | 6,045 | ( 24.1 ) | 4,212 | ( 24.4 ) |
| Did previously but quit after learning of pregnancy | 9,426 | ( 13.0 ) | 1,816 | ( 14.0 ) | 2,454 | ( 14.2 ) | 3,145 | ( 12.6 ) | 2,011 | ( 11.7 ) |
| Currently smoking | 2,517 | ( 3.5 ) | 628 | ( 4.8 ) | 685 | ( 4.0 ) | 801 | ( 3.2 ) | 403 | ( 2.3 ) |
| **Employed, n (%)** |  |  |  |  |  |  |  |  |  |  |
| No | 37,404 | ( 51.5 ) | 6,659 | ( 51.2 ) | 8,715 | ( 50.4 ) | 12,883 | ( 51.4 ) | 9,147 | ( 53.0 ) |
| Yes | 35,220 | ( 48.5 ) | 6,338 | ( 48.8 ) | 8,587 | ( 49.6 ) | 12,175 | ( 48.6 ) | 8,120 | ( 47.0 ) |
| **Infant sex, n (%)** |  |  |  |  |  |  |  |  |  |  |
| Boy | 37,109 | ( 51.1 ) | 6,677 | ( 51.4 ) | 8,781 | ( 50.8 ) | 12,786 | ( 51.0 ) | 8,865 | ( 51.3 ) |
| Girl | 35,515 | ( 48.9 ) | 6,320 | ( 48.6 ) | 8,521 | ( 49.3 ) | 12,272 | ( 49.0 ) | 8,402 | ( 48.7 ) |
| **Nursery attendance, n (%)** |  |  |  |  |  |  |  |  |  |  |
| No | 52,804 | ( 72.7 ) | 9,242 | ( 71.1 ) | 12,405 | ( 71.7 ) | 18,254 | ( 72.9 ) | 12,903 | ( 74.7 ) |
| Yes | 19,820 | ( 27.3 ) | 3,755 | ( 28.9 ) | 4,897 | ( 28.3 ) | 6,804 | ( 27.2 ) | 4,364 | ( 25.3 ) |
| **Location where infant sleeps at night, n (%)** |  |  |  |  |  |  |  |  |  |  |
| In parent's bed | 55,757 | ( 76.8 ) | 9,748 | ( 75.0 ) | 13,236 | ( 76.5 ) | 19,442 | ( 77.6 ) | 13,331 | ( 77.2 ) |
| In baby bed in parents' bedroom | 16,395 | ( 22.6 ) | 3,151 | ( 24.2 ) | 3,952 | ( 22.8 ) | 5,463 | ( 21.8 ) | 3,829 | ( 22.2 ) |
| In baby bed in another room | 389 | ( 0.5 ) | 80 | ( 0.6 ) | 100 | ( 0.6 ) | 121 | ( 0.5 ) | 88 | ( 0.5 ) |
| Other | 83 | ( 0.1 ) | 18 | ( 0.1 ) | 14 | ( 0.1 ) | 32 | ( 0.1 ) | 19 | ( 0.1 ) |
| **Birth weight, g** | 3,030 | | 3,012 | | 3,027 | | 3,038 | | 3,036 | |
| **Gestational weeks** | 39.3 | | 39.3 | | 39.3 | | 39.3 | | 39.3 | |
| **Disease, n (%)** | 13,775 | ( 19.0 ) | 2,455 | ( 18.9 ) | 3,331 | ( 19.3 ) | 4,767 | ( 19.0 ) | 3,222 | ( 18.7 ) |

^a^ Dietary intake between learning of pregnancy and second/third trimester.

BMI, body mass index
